# Quantification of Viral RNA and DNA Positive Cells in Tissues From Simian Immunodeficiency Virus/Simian Human Immunodeficiency Virus Infected Controller and Progressor Rhesus Macaques

**DOI:** 10.3389/fmicb.2019.02933

**Published:** 2019-12-20

**Authors:** Bapi Pahar, Dot Kuebler, Terri Rasmussen, Xiaolei Wang, Sudesh K. Srivastav, Arpita Das, Ronald S. Veazey

**Affiliations:** ^1^Division of Comparative Pathology, Tulane National Primate Research Center, Covington, LA, United States; ^2^Department of Biostatistics and Bioinformatics, Tulane University, New Orleans, LA, United States; ^3^Division of Microbiology, Tulane National Primate Research Center, Covington, LA, United States

**Keywords:** DNA, HIV, infection, macaque, RNA, SIV, tissue

## Abstract

Eradication of human immunodeficiency virus 1 (HIV-1) from an infected individual cannot be achieved using current antiretroviral therapy (ART) regimens. Viral reservoirs established in early infection remain unaffected by ART and are able to replenish systemic infection upon treatment interruption. Simian immunodeficiency virus (SIV) infected macaque models are useful for studying HIV pathogenesis, treatments, and persistent viral reservoirs. Here, we used the SIV macaque model to examine and quantify RNA and DNA positive cells in tissues from macaques that control viral replication (controllers) and those that have persistently high plasma viremia (progressors). A positive correlation was detected between tissue RNA+ cells and plasma viral load in both mesenteric lymph node (LN) and spleen. Similarly, a positive correlation also observed between DNA+ cells and plasma viral load in ileum and jejunum. Controllers had a lower frequency of both RNA and DNA+ cells in several tissues compared to progressors. However, DNA+ cells were prevalent in mesenteric LN, inguinal LN, colon, midbrain, and bone marrow tissues in both controller and progressors. Organized lymphoid tissues of LNs, spleen, and intestine were found as the major tissues positive for virus. Viral RNA and DNA positive cells were detected in brain and thymus in macaques with high plasma viremia and SIV-encephalitis. Both T cells and macrophages were shown to be infected in several tissues, indicating vaccines and ART should be specifically designed to protect these cells in organized lymphoid tissues. These results indicate ART should target infected cells in secondary lymphoid organs to reduce both productively and latently infected cells.

## Introduction

Human immunodeficiency virus (HIV) continues to be a major dreaded disease, as 37.9 million people are living with HIV/AIDS worldwide^1^. Globally, in 2018 alone, an estimated 1.7 million people became newly infected with HIV and 770,000 people died from AIDS-related illness, emphasizing the need for more effective treatments. Administration of combination antiretroviral therapy (cART) has drastically improved the mortality of HIV infected patients, yet it has little impact on eliminating viral reservoirs or in reducing virus-associated immune activation, which is believed to be primarily responsible for continued viral replication, and the acceleration of cellular senescence and aging in HIV disease (Desai and Landay, 2010). The simian immunodeficiency virus (SIV) non-human primate (NHP) models of AIDS are well established, and have been extremely useful in HIV-1 vaccine development and in advancing the understanding of the pathogenesis of AIDS. HIV and NHP models of SIV infection have shown that persistent latent and active reservoirs are established in tissues within days of infection (Chun et al., 1998; Whitney et al., 2014; Ananworanich et al., 2016; Amedee et al., 2018; Bandera et al., 2019). A recent study demonstrated the presence of SIV in CD4+ T cells and macrophages in ART suppressed SIV infected macaques treated with cART 12 days after infection (Abreu et al., 2019). Resting CD4+ T cells are believed to be the major latent cellular reservoirs for HIV. Further, CD4+ cells with stem-cell like properties, resident memory T cells in cervical mucosa, follicular T cells in germinal centers of organized lymphoid tissues, monocytes/macrophages, dendritic cells, astrocytes, hematopoietic stem cells, and other progenitor cells have all been shown to be reservoirs for HIV (Zhu et al., 2002; Buzon et al., 2014; Fukazawa et al., 2015; Garcia et al., 2017; Zaikos et al., 2018; Bandera et al., 2019; Cantero-Perez et al., 2019). Major HIV tissue reservoir includes lymph node (LN), spleen, gut associate lymphoid tissues, brain, lung, liver, bone marrow, reproductive tissues and adipose tissues (Chun et al., 1997; Blankson et al., 2002; Marcello, 2006; Couturier and Lewis, 2018), thus virus rebounds within days to a few weeks after ART interruption. Cells latently harboring proviral DNA allow the virus to persist indefinitely in tissues, which may eventually be triggered to produce bursts of viral replication during periods of immune activation and in the absence of ART. Further, these latently infected cells are not recognized or eliminated by the cytolytic T cells. The half-life of latently infected cells has been estimated to be 44 months on treatment, suggesting that virus cannot be eliminated within the lifetime of infected individuals (Siliciano et al., 2003; Strain et al., 2003; Crooks et al., 2015; Bruner and Cohn, 2019). Moreover, the rare occurrence of genetically intact provirus (1 in 100,000) in circulating CD4+ T cells (Bruner and Cohn, 2019) and the lack of cellular markers to distinguish HIV-infected and uninfected cells are all major barriers for establishing an effective cure strategy for HIV. A minor population of HIV infected patients known as long term non-progressors (LTNPs) have been shown to naturally control plasma viral loads (pVL) and maintain stable CD4+ T cell counts for years in the absence of ART. However, the mechanism behind this lack of progression to disease remains elusive. Here, we used the NHP-SIV model to compare viral infected cells in macaques that spontaneously control viral replication to those that have persistently high viremia. Although most Indian-origin rhesus macaques (RMs) progress to disease when infected with SIV_MAC_, a fraction (∼20%) of Chinese origin macaques spontaneously control viremia and become LTNP. In these LTNP Chinese macaques, virus has been detected in the large intestine without any significant changes in immune activation when compared to progressors (Ling et al., 2010). Although several lymphoid tissues have been shown to harbor latently infected cells, we hypothesize that differences in the distribution of viral infected cells exist between animals who control viral replication such as LTNP and those that progress to simian AIDS. In this study, we have quantified SIV RNA and DNA positive cells in several tissues from progressing and non-progressing macaques (Chinese and Indian origin) to determine the major infected tissues harboring SIV/SHIV RNA and DNA positive cells.

## Materials and Methods

### Ethics Statement

In this study, 32 adult Indian (IND) or Chinese (CHI) RMs (*Macaca mulatta*) were screened and shown to be negative for SIV, HIV-2, type-D retrovirus and simian T-cell leukemia virus 1 infection at the beginning of the study (Table 1). All animals were housed and maintained at the Tulane National Primate Research Center (TNPRC) throughout the study. The study was approved by the TNPRC Institutional Animal Care and Use Committee (IACUC) and was conducted within the guidelines of the United States Public Health Service Policy and the *Guide for the Care and Use of Laboratory Animals* (National Research Council, 2011). The TNPRC is fully accredited by the Association for Assessment and Accreditation of Laboratory Animal Care (AAALAC) International. Once infected, animals were housed in Animal Biosafety Level 2 indoor housing with a 12:12-h light/dark cycle, relative humidity of 30–70%, and a temperature of 64–72°F. Water was available *ad libitum* and a standard commercially formulated NHP diet was provided daily and supplemented with fresh fruit and/or forage material no fewer than three times per week as part of the behavioral management program. Animals were housed in stainless steel cages (Allentown, Inc., Allentown, NJ, United States) sized in accordance or in excess of the U.S. Department of Agriculture (USDA) regulations; each cage contained a perch, portable enrichment toy, and a forage board for feeding enrichment. All animal procedures including virus administration, sample collection, and euthanasia were carried out under the direction of TNPRC veterinarians.

**TABLE 1:** List of rhesus macaques examined.

Animal number	Indian/Chinese^a^	Age at inoculation (year)	Sex^b^	Virus	Days of infection	Dosage (TCID_50_ or vRNA copies)^c^	Route^d^	Terminal plasma viral load (RNA copies/ml)	Cause of death
AL78	CHI	5.2	F	SHIV162P3	204	300	IVAG	<125	Euthanasia
BA69	IND	9.6	F	SHIV162P3	175	300	IVAG	<125	Euthanasia
BA11	IND	10.6	F	SHIV162P3	175	300	IVAG	<125	Euthanasia
BN81	IND	5.9	F	SHIV162P3	520	5000	IR	<125	Intestine Torsion
CR02	CHI	6.2	F	SHIV162P3	129	300	IVAG	699	Euthanasia
BN87	IND	6.7	F	SHIV162P3	175	300	IVAG	2425	Euthanasia
R870	IND	11.3	F	SHIV162P3	175	300	IVAG	181619	Euthanasia
V120	IND	9.7	F	SHIV162P3	175	300	IVAG	916	Euthanasia
GE64	CHI	5.0	F	SHIV162P3	129	500	IVAG	1123	Euthanasia
DE99	IND	4.9	M	SIV_MAC_239	119	100	IV	43244100	Euthanasia due to poor prognosis
DG96	IND	4.7	M	SIV_MAC_239	147	100	IV	12411500	Euthanasia due to poor prognosis
J541	CHI	10.8	F	SIV_MAC_239	556	100	IV	2057775	Colitis
CR83	IND	3.1	F	SIV_MAC_239	616	100	IV	5458	Euthanasia
DD88	IND	2.2	F	SIV_MAC_239	385	100	IV	921755	Euthanasia due to poor prognosis
CA84	IND	4.5	M	SIV_MAC_239	628	100	IV	8285600	AIDS
DD95	IND	2.6	M	SIV_MAC_239	468	100	IV	1819200	Euthanasia due to poor prognosis
V515	CHI	4.2	F	SIV_MAC_239	668	100	IV	183648	*Mycobacterium avium intracellulare*
AL26	CHI	3.1	M	SIV_MAC_239	685	100	IV	949800	Interstitial Pneumonia
P772	CHI	6.5	F	SIV_MAC_239	819	100	IV	2269180	*Pneumocystis carinii* infection
L395	CHI	8.9	F	SIV_MAC_239	1313	100	IV	667	Amyloidosis
V542	CHI	4.1	M	SIV_MAC_239	1845	100	IV	220	Pneumonia
I553	IND	12.2	F	SIV_MAC_239	655	100	IV	1895400	*Pneumocystis carinii* infection
T798	IND	6.3	M	SIV_MAC_239	962	100	IV	85463	*Mycobacterium avium intracellulare*
FA97	CHI	11.8	M	SIV_MAC_239	316	194,981^∗^	IV	3985200	AIDS/CMV infection
BE86	IND	5.9	F	SIV_MAC_251	837	100	IV	76639	*Mycobacterium avium intracellulare*
DG55	IND	3.7	M	SIV_MAC_251	78	100	IR	413577	Euthanasia
CF35	IND	4.2	M	SIV_MAC_251	93	200	IR	2183900	Euthanasia
DB53	IND	4.9	M	SIV_MAC_251	79	200	IR	127999	Euthanasia
DT83	CHI	8.1	F	SIV_MAC_251	456	200	IR	<125	Dehydration
BE64	IND	3.3	F	SIV_MAC_251	752	3,300,000^∗^	IV	265116	Pericarditis
BE65	IND	3.3	M	SIV_MAC_251	1087	3,300,000^∗^	IV	85463	*Mycobacterium avium intracellulare*
CA66	CHI	5.7	M	SIV_MAC_251	746	200	IR	<125	Generalized amyloidosis

*^*a*^IND and CHI denote Indian and Chinese, respectively. ^*b*^F and M denote female and male respectively. ^*c*^All values are expressed as the TCID50, except values denoted by an asterisk (^∗^), which are vRNA copies. TCID_50_ and vRNA represent as tissue culture infectivity dose at 50% and viral RNA respectively. ^*d*^IR, IV, and IVAG denote intrarectal, intravenous, and intravaginal route, respectively. CMV denotes cytomegalovirus.*

### Animals and Tissue Sampling

A total of 32 RMs of both sexes between 2.2 and 12.2 years of age were grouped into four groups based on their terminal pVL. Animals were infected with either SHIV162P3, or SIV_MAC_251 or SIV_MAC_239 using intravenous (IV), intrarectal (IR) or intravaginal (IVAG) routes as listed in Table 1. At necropsy, peripheral blood and tissues including midbrain, mesenteric LN (Mes. LN), inguinal LN (Ing. LN), axillary LN (Ax. LN), spleen, bone marrow, colon, ileum, jejunum, and thymus were collected and processed as both formalin fixed, paraffin embedded tissues and snap frozen in OCT for *in situ* hybridization (ISH) and immunohistochemistry, respectively.

### Plasma Viral Load Quantification

Plasma viral load quantification was performed by bDNA signal amplification assay (Siemens Diagnostics, United States) with a lower limit of detection of 125 SIV/SHIV RNA copies/mL of plasma (Pahar et al., 2007, 2016).

### SIV RNA *in situ* Hybridization

SIV RNA ISH was performed on tissue sections as described previously (Borda et al., 2004; Wang et al., 2007; Ahsan et al., 2013; Pahar et al., 2017). Formalin-fixed, paraffin-embedded tissue sections were de-paraffinized overnight at 60°C, then dehydrated by xylene washes followed by re-hydration with alcohol and DEPC water. Antigen retrieval was performed by treating tissue slides with steam in 0.01 M citrate buffer pH 6.0 using a conventional microwave. A 60-min pre-hybridization incubation was performed followed by an overnight incubation at 45°C in hybridization buffer with SIV RNA digoxigenin labeled probe comprising essentially the entire SIV genome (Lofstrand Labs Ltd., Gaithersburg, MD, United States). The next day, slides were washed with SSC buffer followed by blocking with protein blocker (Dako, Santa Clara, CA, United States) for 1 h. The slides were further incubated overnight with appropriately diluted anti-digoxigenin alkaline phosphatase antibody (Roche Diagnostics, Indianapolis, IN, United States) in dark humidified chambers at 4°C. On day three, after two washes with post-hybridization buffer, the slides were developed with NBT/BCIP (Roche) substrate. For each run, known positive and negative LN controls sections were included to validate staining. An average of 10–15 fields (200× magnification) were used in each of the slides to quantify SIV RNA positive cells manually using SPOT3 live imaging software (Diagnostic Instruments, Sterling Heights, MI, United States). The sites for all tissue evaluations were selected randomly from each tissue and counted by two different individuals blinded to the samples to avoid bias. The quantification of RNA positive cells was presented as the number of infected cells/mm^2^ of tissue.

### SIV DNA *in situ* Hybridization

Amplification of the LTR, Gag, and Pol region of the p239SpSp5′ plasmid DNA (NIH AIDS Research and Reference Reagent Program) was performed utilizing 9 primer pairs designed to span the desired regions. The template DNA (50 ng) and 200 nmoL of each primer were added to a PCR mixture tube containing 2.5 U of Taq DNA polymerase, 200 μM each dTP, dCTP, dGTP, 130 μM dATP, 70 μM DIG-UTP (Roche), 10 mM Tris–HCl (pH 8.3), 40 mM KCl, 1.5 mM MgCl_2_. The volume was adjusted with distilled water to 25 μL. The reaction mixture was subjected to denaturation at 95°C for 1 min followed by 40 cycles of 95°C for 1 min, 60°C for 1 min, and 72°C for 1 min, and then a final extension step of 72°C for 7 min was done. Labeled amplicons varied in length, averaging 670 nucleotides long. ISH was performed as previous described (Wang et al., 2007) using the PCR generated non-radioactive digoxigenin-labeled probe. In brief, slides were deparaffinized in xylene, and rehydrated, followed by antigen retrieval with steam (citrate buffer). The sections were first treated with RNAse followed by incubation at 37°C overnight with the DNA probe, after which the hybridized probe was detected with Anti-DIG-POD followed by NBT/BCIP (Roche) for visualization. The probe was tested on negative control tissue and tissues known to be infected with SIV_MAC_239 and SHIV to determine specificity and efficiency (Supplementary Figure 1). Ten fields (200× magnification) were counted from each slide to quantify SIV DNA positive cells manually using SPOT3 live imaging software. The sites for all tissue evaluations were selected randomly from each tissue and counted by two different individuals blinded to the samples to avoid bias as being performed for RNA stained tissues. The quantification of DNA positive cells was presented as the number of infected cells/mm^2^ of tissue.

### Immunohistochemistry for Detection and Phenotyping of Infected Cells in Tissues

Formalin fixed, paraffin embedded, tissue sections were processed for immunofluorescent staining with one or a combination of primary antibodies as previously described (Wang et al., 2007; Pan et al., 2014; Pahar et al., 2015, 2017). For immunofluorescent staining, tissue sections were stained sequentially for 2–3 colors by first staining with antisense SIV riboprobes. SIV mRNA positive cells were developed by using a 2-hydroxy-3-naphthoic acid-2 phenylanilide phosphate (HNPP) fluorescence detection kit (Roche Diagnostics). SIV RNA stained slides were further stained with any one of the unconjugated primary antibodies (CD3, Ham56, DC-SIGN, or Ki67) sequentially and then incubated with Alexa Fluor 488-conjugated secondary antibodies (1:1000 dilution, Life Technologies, United States) and/or Alexa Fluor 633-conjugated secondary antibodies (1:1000 dilution, Life Technologies) sequentially. After staining, slides were washed, mounted with Prolong^®^ Gold antifade medium (Life Technologies) and scanned for imaging using a TCS SP2 confocal laser scanning microscope (Leica, Germany) equipped with three lasers. ImageJ (version 1.52a; NIH, Bethesda, MD, United States) and Adobe Photoshop CC (20.0.4 release; Adobe, San Jose, CA, United States) was used to assign colors to all four channels collected: HNPP/Fast Red, which fluoresces red when exposed to a 568-nm wavelength laser; Alexa 488 (Life Technologies) fluoresces green; Alexa 633 (Life Technologies) appears blue; and the differential interference contrast (DIC) image is gray scale. Negative control slides were incorporated in each experiment either by omitting the primary antibody or using isotype IgG1 and IgG (H + L) controls (Pan et al., 2012, 2013, 2014; Pahar et al., 2015, 2017). For phenotyping SIV-infected cells, tissues were first processed for SIV RNA ISH and immunohistochemistry for Ham56 (clone Ham56, IgM Kappa; Dako), CD3 (Rabbit anti-human polyclonal; Dako), dendritic cell-specific ICAM-3 grabbing non-integrin (DC-SIGN, clone DCN46, IgG2b kappa; BD Biosciences, San Jose, CA, United States), and Ki67 (clone MIB-1, IgG1 kappa; Dako) by multilabel confocal microscopy as previously described (Pahar et al., 2017).

### Graphics and Statistical Analyses

All data was summarized using descriptive statistics including mean, proportions, and standard deviations. Graphical presentation was performed using GraphPad Prism (version 8.1.2. GraphPad Software, United States). The analysis of variance and Chi-square methods were performed to compare the mean and proportional differences data, respectively. A two-sided 5% significance level was used throughout. The associations between the frequency of RNA or DNA positive cells in tissues and pVL for both SHIV and SIV controllers and progressors was calculated using the Pearson correlation coefficient method. All data analyses were performed using SAS software (version 9.4 in a Windows environment).

## Results

### Terminal Plasma Viral Loads in SIV/SHIV Infected Macaques

All RMs were placed into four groups based on their terminal pVL. The first group, SHIV162P3 controllers included four SHIV162P3 infected animals (3 IND and 1 CHI) with undetectable viremia (<125 copies RNA/mL of plasma) (Table 1). The second group, SHIV162P3 progressors included five SHIV162P3 infected animals (3 IND and 2 CHI) ranging from 699 copies to 1.8 × 10^5^ copies of RNA/mL of plasma. The third group, SIV_MAC_251 controllers included two SIV_MAC_251 infected CHI RMs (DT83 and CA66) with undetectable plasma viral load (<125 copies RNA/mL of plasma). The ultimate fourth group, SIV_MAC_239/251 progressors included 14 IND and 7 CHI RMs that were infected either with SIV_MAC_239 or SIV_MAC_251 and had detactable plasma viral load ranging from 220 copies to 4.3 × 10^7^ RNA copies/mL of plasma (Table 1). No RMs infected with SIV_MAC_239 were found to be controllers based on our criteria in this study. The duration of infection prior to necropsy and tissue collection ranged from 78 days to 1845 days (∼5 years) after SIV/SHIV infection. Viremia ranged in SHIV infected animals from (<125 copies) to 1.8 × 10^5^ RNA copies per mL of plasma at the time of euthanasia irrespective of IVAG or IR challenge (Table 1). Four of the SHIV-infected animals that had undetectable pVL (<125 copies) were considered non-progressors, whereas, only one IND macaque (BN81) was categorized as a LTNP having undetectable viremia for over a year. In contrast, 13 out of 15 RMs infected with SIV_MAC_239 had high terminal pVL ranged from 5.5 × 10^3^ to 4.3 × 10^7^ RNA copies/mL of plasma (Table 1). Only two out of 15 RMs infected with SIV_MAC_239 had <1000 copies of plasma RNA copies/mL of plasma at the time of euthanasia, and both were of Chinese origin. Similarly, 6 out of 8 SIV_MAC_251 infected RMs, had high pVL ranging from 7.7 × 10^4^ to 2.2 × 10^6^ RNA copies/mL of plasma at necropsy. All other RMs that had detectable pVL were considered normal progressors. We did not detect significant differences in terminal pVL between animals infected by different routes of inoculation. The majority of the SIV_MAC_239 infected macaques were euthanized due to AIDS related symptoms or opportunistic infections (Table 1). None of the SHIV infected RMs had detectable pathological findings except for one IND macaque (BN81) that was euthanized due to intestinal torsion which was not considered to be associated with the SHIV infection.

### Quantification of SIV/SHIV RNA Positive Cells in Tissues

**FIGURE 1:**
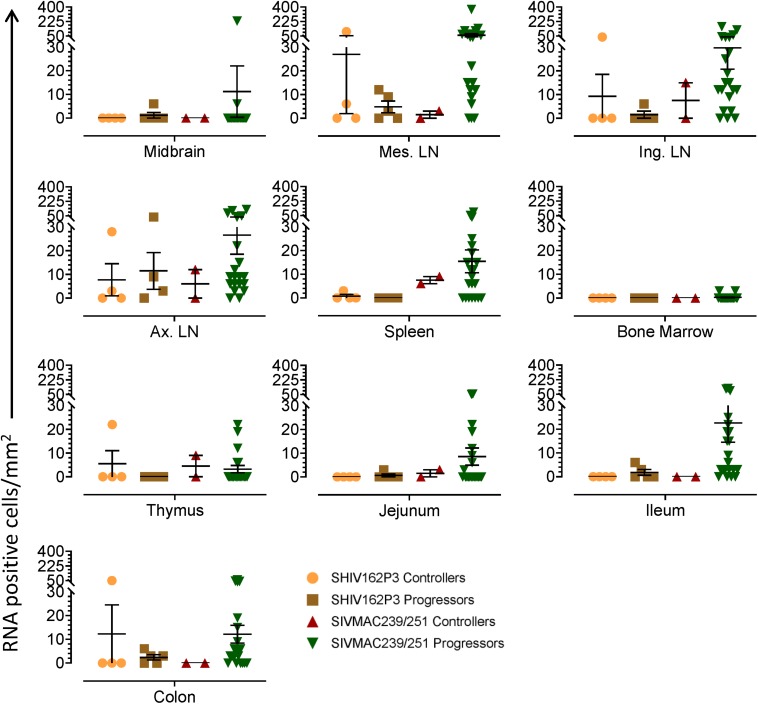
Quantification of RNA positive cells/mm^2^ per section in different tissues from animals with different terminal plasma viral loads are shown. Horizontal lines denoted the mean RNA positive cells (±SEM) for each group of animals for the respective tissue.

**FIGURE 2:**
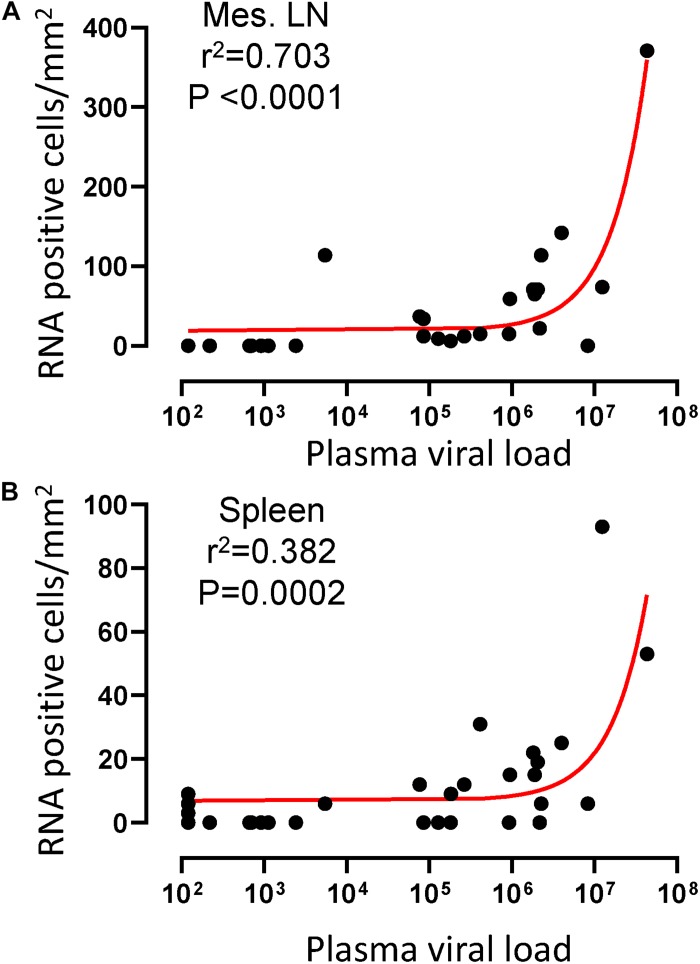
A positive correlation of RNA positive cells and plasma viral RNA copies was shown for both mesenteric lymph node **(A)** and spleen **(B)** tissues. Pearson correlation coefficients analysis of RNA positive cells/mm^2^ and plasma viral RNA load (RNA copies/mL of plasma) from all SHIV/SIV controllers and progressors was performed.

**FIGURE 3:**
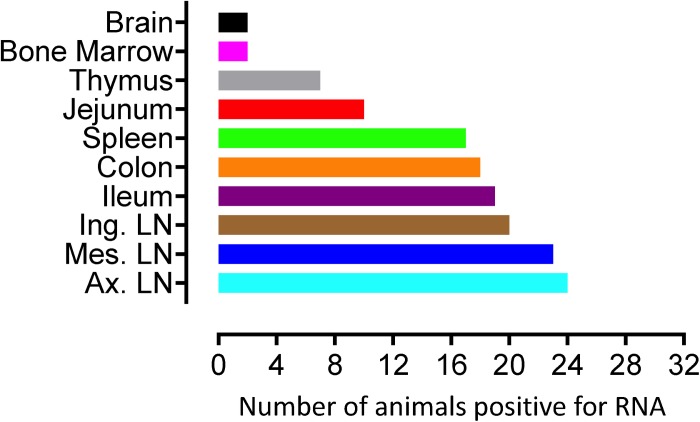
Number of animals with RNA positive cells in each tissue are shown. Ten different tissues in a total of 32 animals were examined. Axillary LN and bone marrow/brain were found to harbor the most, and least number of RNA+ cells, respectively compared to other tissues examined.

To quantify virus infected cells in animals, we performed ISH staining for viral RNA in different tissues and the frequency was quantified as RNA positive cells/mm^2^ of tissue (Figure 1). All animals except BN81 (infected with SHIV) had detectable SIV/SHIV RNA positive cells in at least one of the tissues examined. One SHIV progressor (R870) and two SIV progressors (FA97 and J541) had detectable RNA positive cells in midbrain (Table 1 and Figure 1). One IND SHIV controller macaque (BA69) had higher RNA positive cells in mes. LN, ing. LN, ax. LN and colon tissues compared to other SHIV controllers (Figure 1). Overall in LTNPs with undetectable pVL, RNA positive cells were still detected in ing. LN, ax. LN, spleen, colon, jejunum and thymus (Table 2). Similarly, both the SIV controllers had detectable RNA positive cells in spleen. None of the SHIV/SIV controllers had detectable RNA positive cells in midbrain, bone marrow, and ileum (Figure 1). There were no significant differences in numbers of RNA positive cells between controllers and progressors in any of the tissues examined. Total RNA positive cells/mm^2^ in some tissues correlated with pVL in all animals. Parametric Pearson correlation coefficient of these independent measures demonstrated a highly significant positive correlation of increased pVL and RNA positive cells in mes. LN (*p* < 0.0001, Figure 2A) and spleen (*p* = 0.0002, Figure 2B). However, no significant correlation was detected between RNA positive cells in any other tissues (midbrain, ing. LN, ax. LN, bone marrow, thymus, jejunum, ileum and colon) and pVL. We also compared frequencies of tissues that were positive for viral RNA from each animal. Ax. LN was found to be the most frequent infected tissue as 25 out of 32 animals were found positive for viral RNA (Figure 3). LNs were the most frequent infected tissues for SIV/SHIV RNA followed by ileum, colon, spleen, jejunum, thymus, midbrain and bone marrow (Figure 3).

**TABLE 2:** Summary of viral RNA and DNA positive cells in tissues of macaques spontaneously controlling viremia^#^.

	Brain		Mes. LN		Ing. LN		Ax. LN		Spleen		Bone marrow		Colon		Ileum		Jejunum		Thymus	
	RNA	DNA	RNA	DNA	RNA	DNA	RNA	DNA	RNA	DNA	RNA	DNA	RNA	DNA	RNA	DNA	RNA	DNA	RNA	DNA
AL78	0	0	0	3	0	5	3	0	0	2	0	0	0	0	0	0	0	0	22	0
BA69	0	NE	102	NE	37	NE	28	NE	0	NE	0	NE	49	NE	0	NE	0	NE	0	NE
BA11	0	0	6	11	0	NE	0	NE	3	0	0	0	0	37	0	NE	0	0	0	NE
BN81	0	NE	0	NE	0	0	0	0	0	NE	0	NE	0	NE	0	0	0	NE	0	NE
DT83	0	0	3	1	15	1	12	0	9	0	0	3	0	163	0	0	0	1	1	0
CA66	0	2	0	7	0	14	0	0	6	20	0	0	0	0	0	0	3	3	0	0

*^#^Cells/mm^2^, NE indicates tissue was not examined.*

### Quantification of Viral DNA Positive Cells in Tissues

**FIGURE 4:**
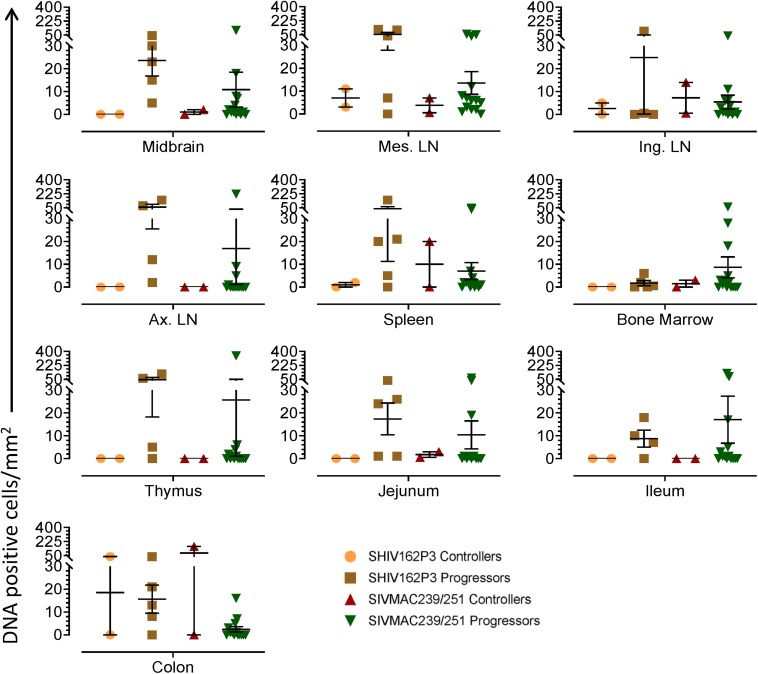
Quantification of DNA positive cells/mm^2^ per section in different tissues from animals with different plasma viral loads are shown. Horizontal lines denoted the mean DNA positive cells (±SEM) for each group of animals for the respective tissue.

**FIGURE 5:**
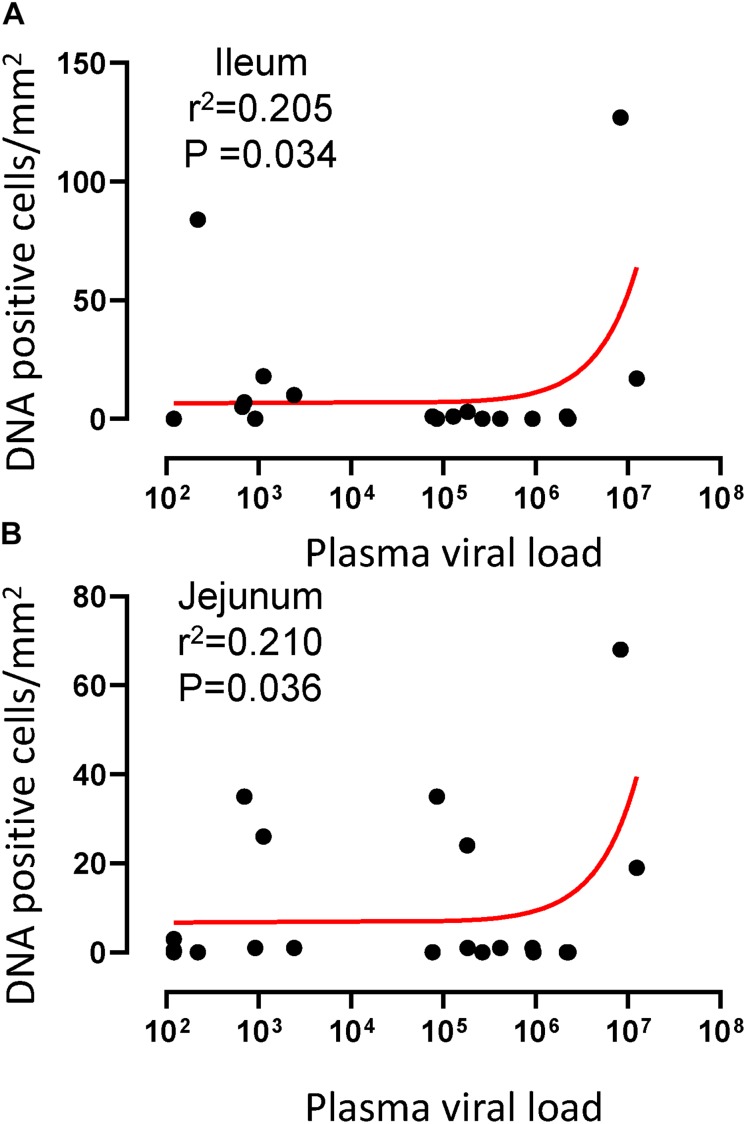
A positive correlation of DNA positive cells and plasma viral RNA copies was shown for both ileum **(A)** and jejunum **(B)** tissues. Pearson correlation coefficients analysis of DNA positive cells/mm^2^ and plasma viral RNA load (RNA copies/mL of plasma) from all SHIV/SIV controllers and progressors was performed.

**FIGURE 6:**
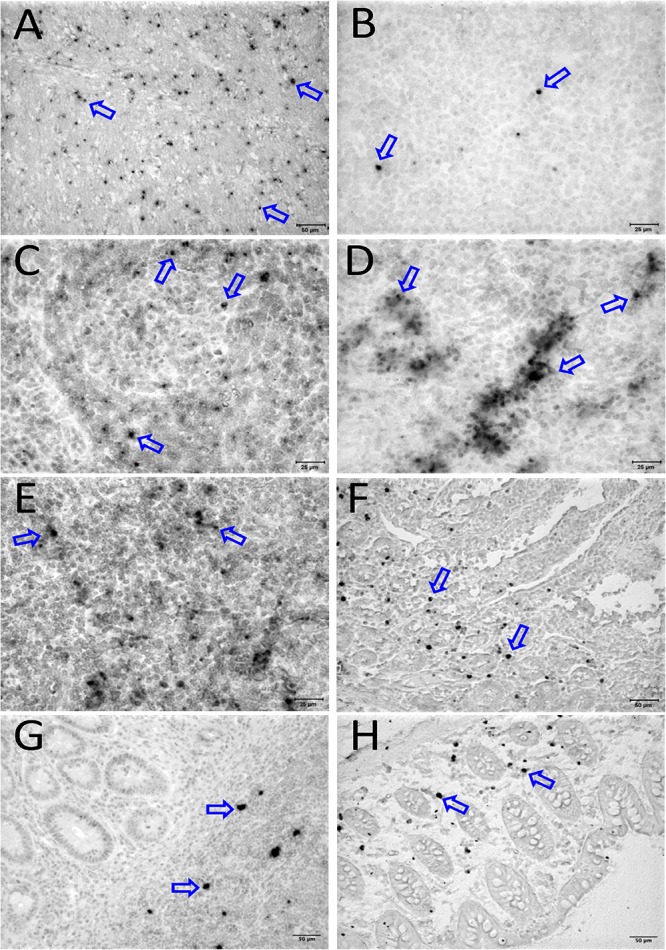
Detection of viral reservoirs in tissues of macaques with high viremia by *in situ* hybridization for viral DNA. SIV DNA positive cells are shown in brown for the brain (CR02, **A**), bone marrow (DD88, **B**), axillary LN (GE64, **C**), mesenteric LN (CR02, **D**), inguinal LN (DG96, **E**), jejunum (CA84, **F**), ileum (DG96, **G**), and colon (R870, **H**). Blue arrows in each figure indicate representative SIV-DNA positive cells.

To quantify the DNA positive cells, we performed ISH staining in tissues from 25 animals (Figure 4). Cells positive for viral DNA were quantified as DNA positive cells/mm^2^ area in tissues as described in methods. All animals except BN81 (infected with SHIV) had detectable DNA positive cells in at least one of the tissues examined. None of the animals with undetectable viremia (<125 copies/mL of plasma) had detectable DNA positive cells in ax. LN, thymus, or ileum, suggesting these may not be the major viral reservoirs for LTNP animals (Figure 4). In contrast, mes. LN followed by ing. LN and colon were found to be major source of viral DNA for several macaques with undetectable pVL and LTNPs. All SHIV progressors and 10 out of 14 SIV progressors had detectable DNA positive cells in midbrain suggesting that the frequency of viral DNA is much higher in brain compared to viral RNA copies in those animals. Similarly, 3 out of 5 and 8 out of 14 macaques from SHIV and SIV_MAC_251/239 progressors, respectively were positive for viral DNA in bone marrow (Figure 4) which was much higher in frequency than RNA positive cells in that tissue. No significant differences in DNA positive cells were detected between controller and progressor groups in any tissues examined. Total DNA positive cells/mm^2^ in each tissue however correlated with pVL for all animals. Parametric Pearson correlation coefficient of these independent measures demonstrated a significant positive correlation with pVL and numbers of DNA positive cells/mm^2^ in the ileum (*p* = 0.036, Figure 5A) and jejunum (*p* = 0.036, Figure 5B). However, no correlation between pVL and tissue DNA positive cells was detected for any other tissue (midbrain, mes. LN, ing. LN, ax. LN, spleen, bone marrow, thymus, or colon). We also quantified the number of animals positive for viral DNA in tissues. Mes. LN was found to be the major infected tissue, where 19 out of 25 animals examined were positive for viral DNA. LNs appeared to be the major infected tissue followed by spleen, midbrain, colon, ileum, bone marrow, jejunum, and thymus. In the sections examined, viral DNA was diffusely distributed throughout the section in several tissues including midbrain (Figure 6A, CR02 with pVL 699 RNA copies/mL of plasma), bone marrow (Figure 6B, DD88 with pVL 9.2 × 10^5^ RNA copies/mL of plasma), ax. LN (Figure 6C, GE64 with pVL 1123 RNA copies/mL of plasma), mes. LN (Figure 6D, CR02), ing. LN (Figure 6E, DG96 with pVL 1.2 × 10^7^ RNA copies/mL of plasma), jejunum (Figure 6F, CA84 with pVL 8.3 × 10^6^ RNA copies/mL of plasma), ileum (Figure 6G, DG96) and colon (Figure 6H, R870 with pVL 1.8 × 10^5^ RNA copies/mL of plasma).

### Quantification of RNA and DNA Positive Cells in Macaques Controlling Viremia

**FIGURE 7:**
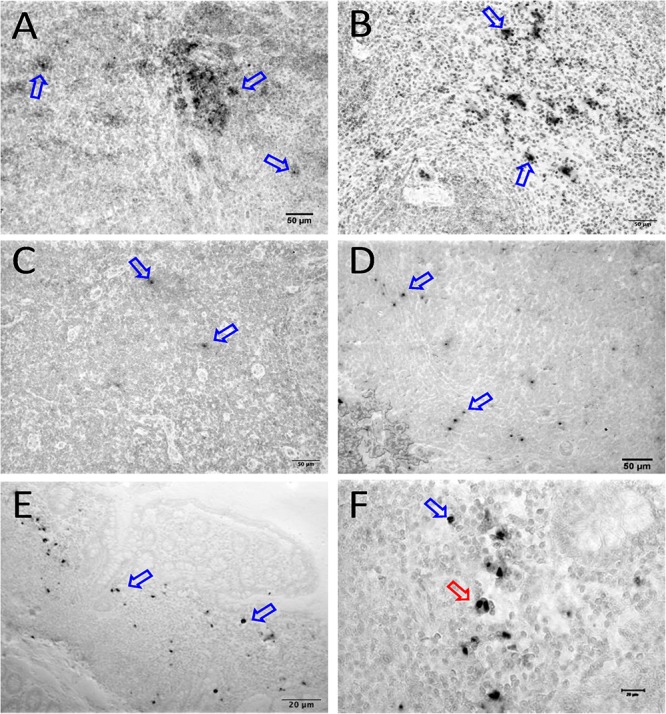
SIV DNA positive cells detected by *in situ* hybridization in LTNP with undetectable viremia are shown in brown for mesenteric LN (BA11, **A**), spleen (CA66, **B**), inguinal LN (AL78, **C**), and colon (DT83, **D**). Similarly, RNA positive cells are shown in brown for colon (BA69, **E,F**) from a LTNP animal with undetectable viremia. All animals had undetectable plasma viral load (<125 copies/ml of plasma) at the time of euthanasia. Blue arrows in each figure indicate representative SIV-DNA/RNA positive cells. Panels **(E,F)** show RNA+ cells in a macaque (BA69), some of which are clearly in the telophase stage of cell division (red arrow). Note, that despite the animals had undetectable plasma viral load, several tissues had productively infected cells as well as DNA positive cells detected at the time of euthanasia.

We quantified RNA and DNA positive cells by ISH from the 6 RMs infected with SHIV or SIV_MAC_251 with undetectable viremia (3 IND and 3 CHI, pVL < 125 RNA copies/mL of plasma) in 10 different tissues except BA69, where only RNA was examined, and none of the tissues were examined for viral DNA+ cells (Figure 7). Similarly, only selected tissues were used to quantify DNA+ cells in animals BA11 and BN81. In the animals examined, SIV DNA+ cells were diffusely distributed throughout tissues examined, especially in mes. LN (Figure 7A, BA11), spleen (Figure 7B, CA66), ing. LN (Figure 7C, AL78), and colon (Figure 7D, DT83). RNA positive cells from colon were shown (Figures 7E,F, BA69), where some of the RNA positive cells were dividing cells (Figure 7F). A summary of the viral reservoirs detected in animals controlling viremia was shown in Table 2. One animal (BN81) had no detectable RNA or DNA in the tissues examined, but as noted, only a limited number of tissues were screened for DNA+ cells in this animal.

### Phenotyping RNA+ Cells in Tissues

**FIGURE 8:**
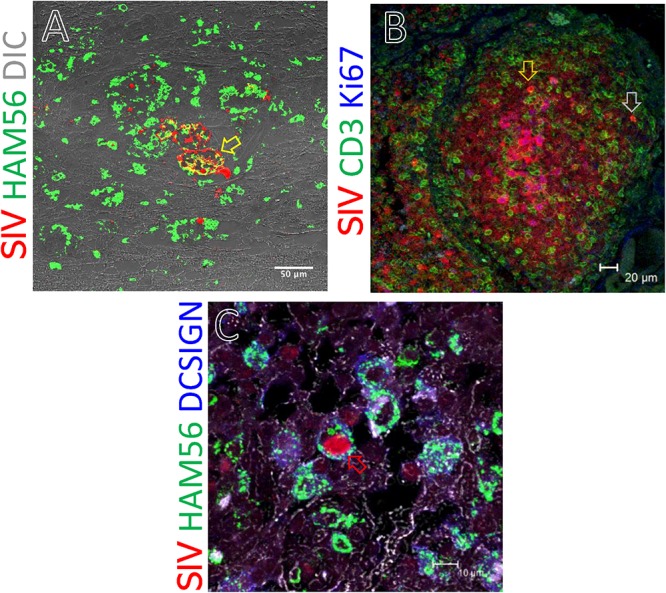
SIV RNA positive cells were detected by *in situ* hybridization and multilabel confocal microscopy in midbrain **(A)**, thymus **(B)**, and inguinal LN **(C)**. Ham56 positive macrophages **(A,C)**, DCSIGN positive dendritic cells **(C)**, and CD3+ T-cells **(B)** are shown in different tissues. Colocalization of Ham56+ and SIV+ cells are shown by yellow arrow. Similarly, distribution of SIV infected cells in proliferating (Ki67^positive^) and resting (Ki67^negative^) cells was shown by orange and gray arrow, respectively. Colocalization of Ham56, DC-SIGN and SIV+ cells are shown by red arrow.

We assessed the phenotype of SIV RNA+ cells in midbrain, thymus, ing. LN, colon, ax. LN, and spleen by multilabel confocal microscopy. As shown in Figure 8, T-cells (CD3+), macrophages (HAM56+), and dendritic cells (CD209, DC-SIGN+) were all shown to harbor SIV RNA (Figure 8). The midbrain contained multiple multinucleated giant cells infected with SIV surrounded by uninfected macrophages. T cells were the most frequently infected cells but rare DC-SIGN positive cells were occasionally found, mostly in the T-cell zones of LN (Figure 8C) and spleen (data not shown). The identification of RNA + HAM56 + DC-SIGN+ cells could either be actively replicating virus infected macrophages that have engulfed virus or virus infected cells (Figure 8C). Several HAM56+ cells (macrophages) were detected in the colon lamina propria, however, very few cells colocalized with SIV RNA (data not shown). In thymus SIV infected CD3+ T cells were also detected (Figure 8B). The majority of SIV RNA+ cells were found to be non-proliferating (Ki67^negative^) in this study but several proliferating (Ki67^positive^) cells were detected in thymus (Figure 8B).

### Differences in Levels of Infected Cells Between Indian and Chinese Origin Macaques

**FIGURE 9:**
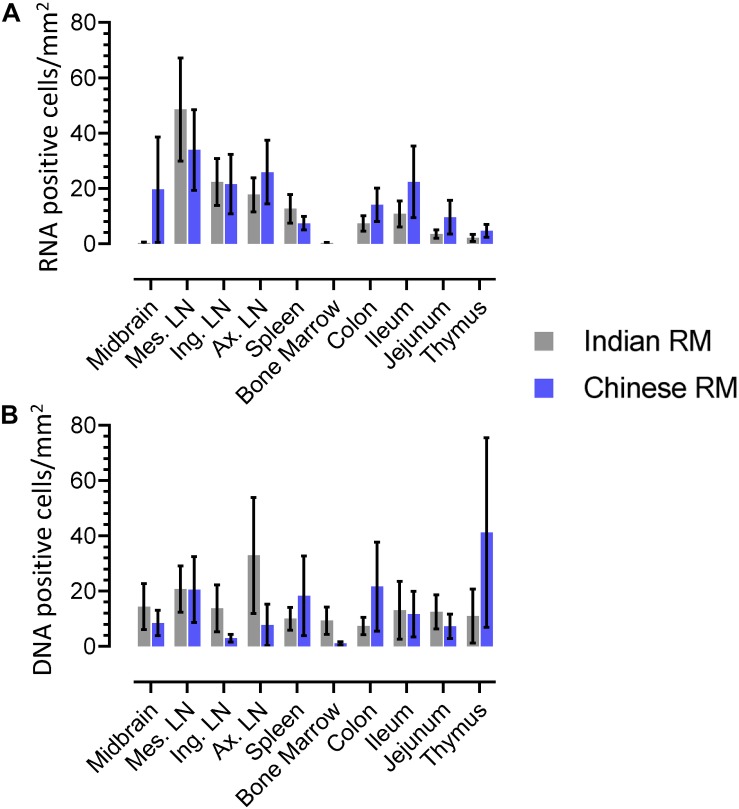
Quantification of RNA **(A)** and DNA **(B)** positive cells in different tissues from Indian and Chinese animals were shown. No statistically significant differences in RNA/DNA+ cells exist between Indian and Chinese animals in each tissue when analyzed using analysis of variance and Chi-square methods. The horizontal bars denoted the mean RNA or DNA positive cells (±SEM) for each group and for the respective tissue.

We quantified RNA and DNA+ cells from twenty IND and twelve CHI RMs for each tissue (Figure 9). Overall, higher numbers of RNA+ cells were detected in both IND and CHI RMs compared to DNA+ cells in LN tissues. DNA positive cells in brain and bone marrow were detected more frequently in IND compared to CHI macaques, however, the differences were not statistically significant. Similarly, increased DNA+ cells were detected in the thymus of CHI compared to IND macaques, but again the differences were not significant. No significant differences in RNA/DNA+ cells were observed between IND and CHI RMs for the other tissues examined.

## Discussion

Combination ART has drastically reduced the mortality rate and improved the life expectancy of HIV infected patients. However, despite years of treatment and years of undetectable plasma viremia in treated patients, cART is still not able to eliminate reservoirs or cure HIV infection. Recent studies with SIV-infected RMs and HIV patients treated with cART showed the presence of both viral RNA and DNA+ cells in several lymphoid tissues indicating long term cART fails to eliminate viral reservoirs in tissues (Estes et al., 2017). Further, most new treatment strategies aimed at reducing viral reservoirs have failed to eliminate actively replicating and latently infected proviruses from tissues (Chun and Fauci, 2012). In addition, cART treated HIV patients also have a higher incidence of age-related diseases than uninfected individuals (Pathai et al., 2014). Interestingly, a small percentage of HIV+ patients spontaneously control pVL below levels of detection, without the administration of cART, although these LTNPs represent less than 1% of the HIV-1 infected population (Blankson, 2010; Mandalia et al., 2012; Imami et al., 2013). Comparing the quantity and distribution of persistent viral reservoirs in tissues from HIV LTNPs compared to HIV progressors may lead to improved treatment and cure strategies. However, recessed tissues, including brain, intestine, bone marrow, etc., are difficult to sample in humans. Thus the SIV infected RM model is the premier model for studying the immunopathogenesis of HIV infection, as well as studying viral reservoir in progressors and LTNPs (Heise et al., 1993; Veazey et al., 1998; Shedlock et al., 2009; Ling et al., 2010; Zaragoza et al., 2011; Pan et al., 2012, 2013, 2014; Pahar et al., 2015).

Here, we showed lower pVL in the majority of RMs infected with SHIV162P3 compared to macaques infected with SIV_MAC_251 or 239, which correlates with lower levels of RNA+ as well as DNA+ levels in SHIV infected animals. However, DNA+ cells persisted, and were prevalent in mes. LN, ing. LN, colon, midbrain and bone marrow tissues in both non-progressors and progressor animals. Mesenteric LN was found to be the major source of viral DNA in macaques with undetectable pVL and LTNPs, which supports previous reports where mes. LN was shown to be the major site of persistent viral reservoir in the presence of cART (North et al., 2010; Horiike et al., 2012; Siddiqui et al., 2019). LNs were found to harbor RNA and DNA+ cells in the majority of the infected animals compared to intestinal tissues. However, only one section of intestinal tissue from each organ was examined, and since the intestinal tract is among the largest organs in the body, single sections may not be representative of the entire tissue reservoir. Organized lymphoid tissues (LN, ileum, colon) are clearly the major tissue for viral RNA and DNA+ cells, likely because virus dissemination is mainly mediated by infected lymphocytes, macrophages and/or dendritic cells that normally re-circulate through organized lymphoid tissues as part of their normal homing process (Moll, 2003). Here, we also show that the large intestine (colon) consistently contains detectable RNA and DNA+ cells, contains more organized lymphoid tissues than the small intestine, and is likely the major persistent viral reservoir in macaques spontaneously controlling viremia in the majority of infected animals, which is also consistent with previous findings of macaques controlling viremia through cART (Van Marle et al., 2007; Ling et al., 2010). A positive correlation between tissue RNA+ cells and plasma viral load in both mes. LN and spleen suggests that these lymphoid tissues may be more exposed to viruses from the gastrointestinal tract compared to other tissues during high SIV/SHIV plasma viral load. Similarly, a positive correlation between DNA+ cells and pVL in ileum and jejunum suggests that perhaps during peak viremia, viral RNA is transcribed to DNA more often in those tissues resulting in larger reservoirs in these tissues compared to other tissues. The presence of RNA+ cells in tissues does not differentiate actively replicating virus or cells with virus that might have reactivated from latency. Similarly, our assay for DNA+ cells in tissues can not discriminate between cells that harbor latent provirus or replication competent virus.

SIV infected T-cells, HAM56+ macrophages, and DC-SIGN (a marker of dendritic cells) were detected in several tissues, demonstrating that all of these cells contribute to viral persistence and even viral replication which is also in agreement with other reports (Wang et al., 2010; Ribeiro Dos Santos et al., 2011; Soulas et al., 2011; Nowlin et al., 2015; Pahar et al., 2017). Although SIV-infected proliferating cells were frequently detected, the majority of infected cells were Ki67^negative^, indicating both proliferating and non-proliferating cells serve as source for harboring infection. However, we did detect significant numbers of RNA+ cells that were clearly dividing (Figure 7E), lending support to the theory that at least some of the viral reservoir persists through cellular division resulting in clonal expansion of virus (Murray et al., 2016). Several studies have documented that resting memory CD4+ T cells are infected with SIV during acute infection (Zhang et al., 2004; Li et al., 2005). It is believed that SIV infected cells are initially infected when the cells are activated or proliferating, and later convert to resting, non-proliferating cells and remained persistent as latently infected cells in tissues. Notably, bone marrow and brain had the least residual infected cells, especially in animals controlling viremia, indicating those tissues play a minimal role as a source for harboring virus. Other tissues such as reproductive organs, liver, breast tissues, and adipose tissues have been described as potential reservoirs for infection (Svicher et al., 2014; Damouche et al., 2015) but these tissues were not examined in this study.

Both IND and CHI RMs are used for HIV/SIV pathogenesis, therapeutics and vaccine research due to their susceptibility to SIV/SHIV infection through different routes of inoculation (Joag et al., 1994; Mellors et al., 1996; Smith et al., 1999; Lifson et al., 2001; Ling et al., 2002; Zhou et al., 2013). Although the same genus and species, CHI macaques are more genetically diverse than IND macaques, and some (20–30%) have significantly lower viral set points, generate strong antibody responses, have reduced induction of type I IFN responses, lower levels of immune activation, and reduced mitochondrial antiviral signaling proteins compared to IND RMs, due to still unknown differences in physiological and genetical parameters (Viray et al., 2001; Ling et al., 2002; Doxiadis et al., 2003; Campillo-Gimenez et al., 2010; Zhou et al., 2013; Zhang et al., 2018). However, we did not observe any significant differences in either RNA or DNA positive cells in tissues between these animals, which suggests that despite their differences in levels of control of viremia, or genetic differences, both IND and CHI origin macaques show similar to equal proportions of viral infected cells in tissues, indicating their suitability as animal models for LTNPs and HIV cure strategies. Similarly, we did not observe any significant difference in either RNA or DNA positive cells in tissues when animals were grouped by sex (male or female), viral dose, or route of virus exposure in our study.

Overall, we have shown that non-progressor animals having a lower frequency of both RNA and DNA+ cells in several tissues compared to progressors. In general, organized lymphoid tissues (LNs and colon) were shown to be the major persistent reservoirs for viral RNA and DNA+ cells in animals spontaneously controlling infection. Therefore, ART should be designed to penetrate organized lymphoid tissues so that it can target all tissue-resident infected cells to reduce the burden of both latently and productively infected cells. Notably, RNA+ cells were frequently detected in organized lymphoid tissues of almost every animal, even those spontaneously controlling infection with undetectable pVL. This study has caveats and limitations including the lack of inclusion of reproductive tissues in our tissue list and the sensitivity of the assays. A more sensitive and specific next-generation ISH approach has been developed for the detection of viral RNA and DNA+ cells using RNAscope and DNAscope probes, respectively (Deleage et al., 2016) that can provide more detailed information about the actively replicating and latently infected cells in these tissues but since resources were limited for this study we used our standard ISH techniques for detecting virus in tissues.

In recent years, several approaches have been proposed and tested to eliminate latent viral reservoirs, including lymphocyte ablation by irradiation followed by bone marrow transplantation with CCR5 deletion of stem cells, gene editing technology to disrupt the HIV genome, and “Shock and Kill” strategies to reactivate latent HIV followed by immune clearance by cART (Chun et al., 2015; Elsheikh et al., 2019). So far, these approaches have shown negligible success in clearing latent HIV reservoirs, and failure to generate effective host immune responses. There are several new strategies and compounds under investigation that may either block, or eliminate latent reservoirs from organized lymphoid tissues in combination with ART, especially those designed to block lymphoid homing, or eliminate infected reservoir cells from LNs by enhancing cellular and humoral immune responses in tissues (Riley and Montaner, 2017). Undoubtedly, more studies are needed to understand the mechanism of HIV latency and better tools to identify and eradicate latently infected cells from tissues.

## Data Availability Statement

The datasets generated for this study are available on request to the corresponding author.

## Ethics Statement

The animal study was reviewed and approved by the Tulane Institutional Animal Care and Use Committee.

## Author Contributions

BP designed the experiments and wrote the manuscript. DK, AD, and BP performed all the animal experiments. BP, AD, and RV performed the data analysis. TR helped in making the DNA probe. XW helped with the RNA ISH assay. SS performed the statistical analyses. RV helped in experiment design and writing manuscript. All authors have given approval to the final version of the manuscript.

## Conflict of Interest

The authors declare that the research was conducted in the absence of any commercial or financial relationships that could be construed as a potential conflict of interest.

## Footnotes

1 https://www.who.int/hiv/data/en/
