# Supplementary material for: Quantification of Viral RNA and DNA Positive Cells in Tissues From Simian Immunodeficiency Virus/Simian Human Immunodeficiency Virus Infected Controller and Progressor Rhesus Macaques
Source: Front Microbiol. 2019 Dec 20;10:2933. doi: 10.3389/fmicb.2019.02933 (PMC6933296; doi:10.3389/fmicb.2019.02933)
Supplement: Supplementary file 1 [file Data_Sheet_1.PDF]

Supplemental Figure 1. Representative immunohistochemistry figures from DNA ISH experiment. (A-C) DNA-ISH staining from three SIV negative, normal rhesus macaque as negative control.(D-E) DNA-ISH staining from two SIV infected animals as positive control. Blue arrows show the positive cells. (F-H) DNA ISH staining from SIV infected animals with no probe.

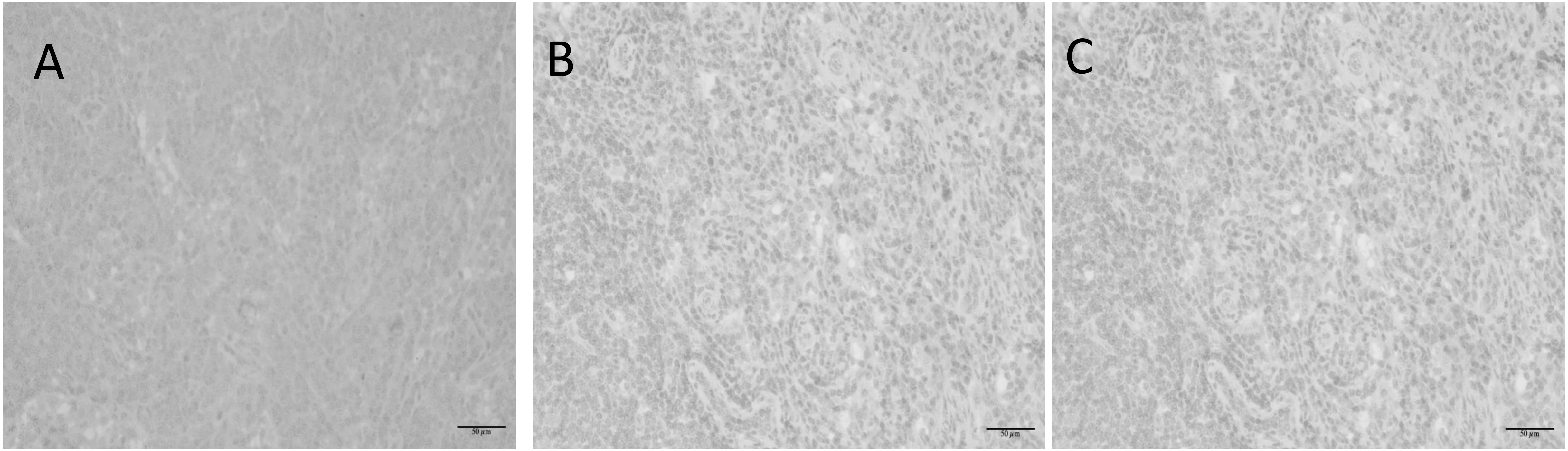

(A-C): Inguinal LN with DNA probe from three SIV uninfected, normal macaque

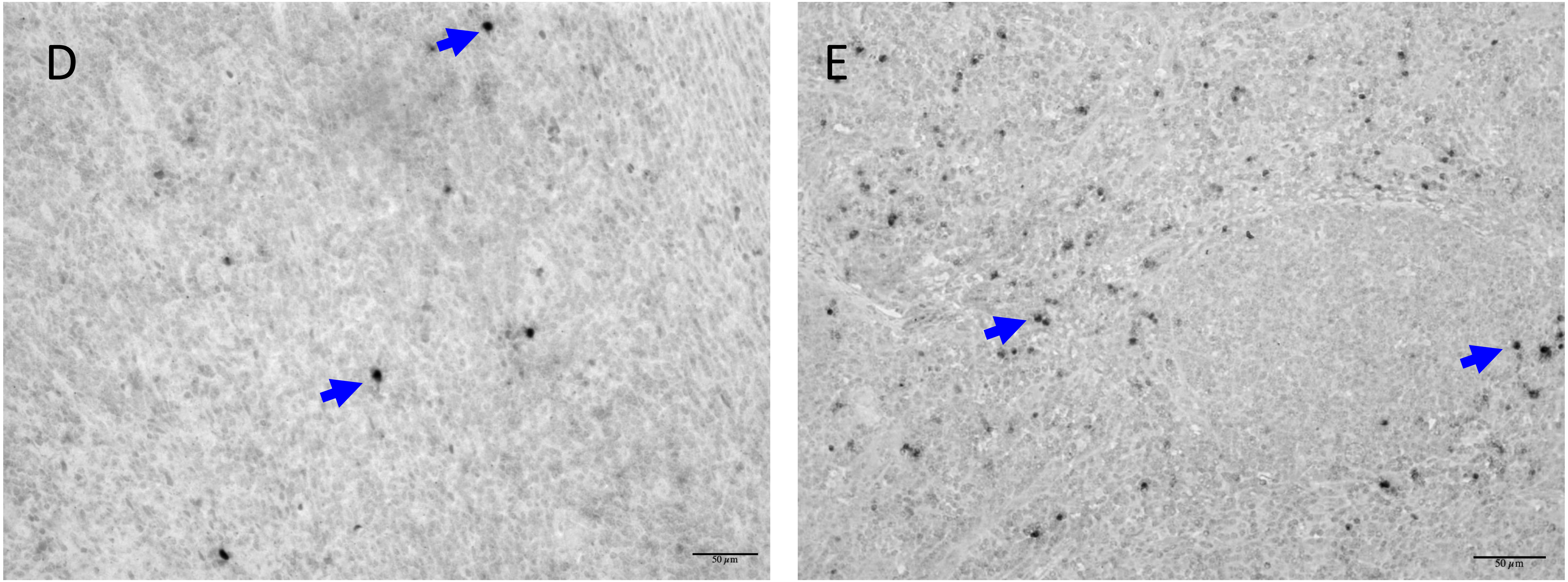

(D and E): Inguinal LN with DNA probe from two SIV infected macaque

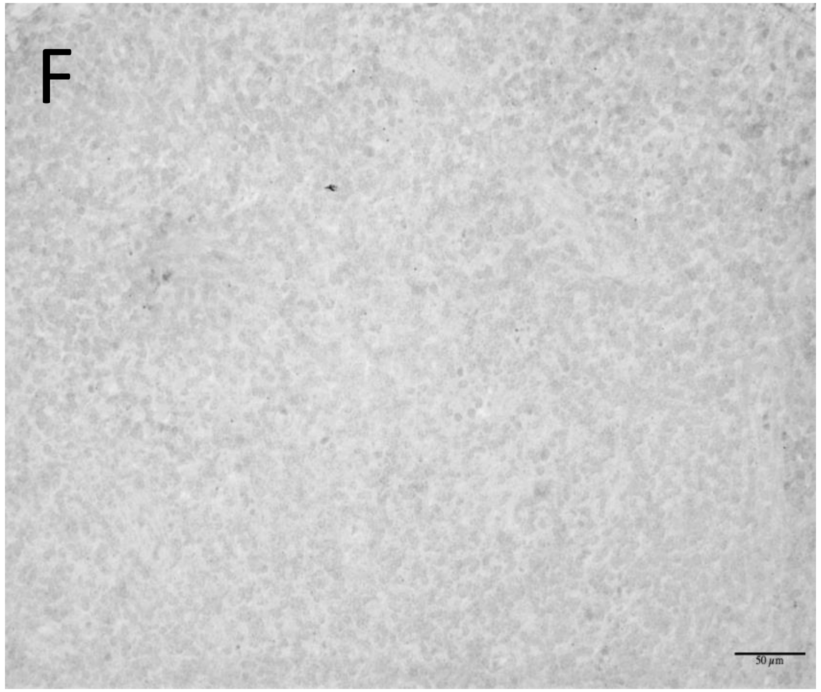

DG96, Inguinal LN, No probe

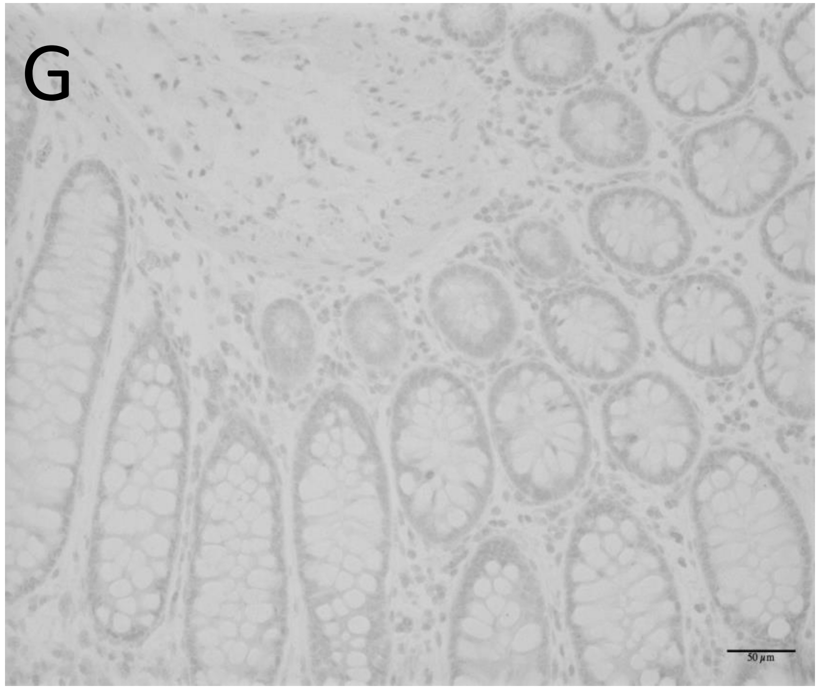

DE99, Jejunum, No probe

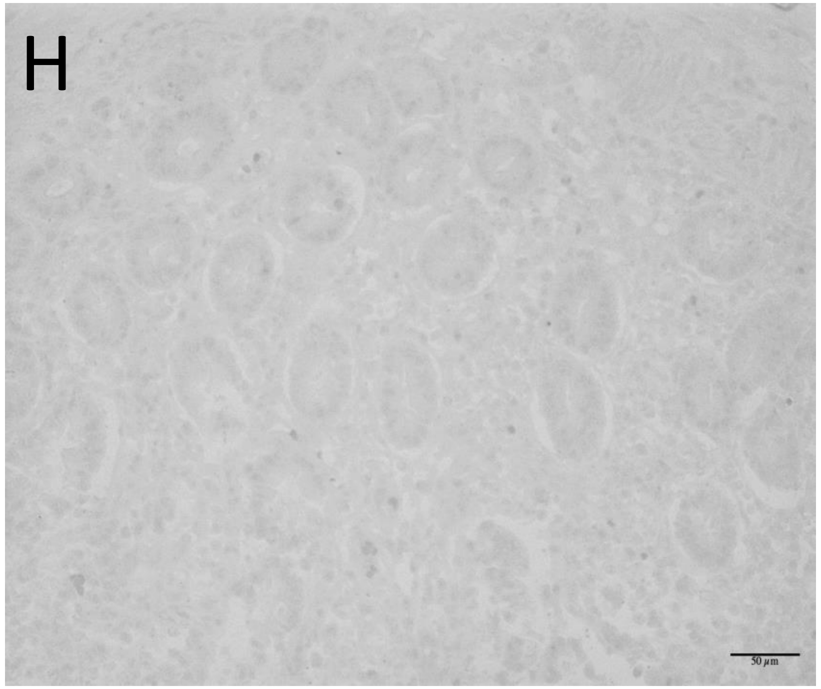

DG96, Ileum, No probe

(F-H): DNA ISH staining in tissues from SIV infected macaque with no probe
